# Invasive lobular carcinoma of the breast presenting as retroperitoneal fibrosis: a case report

**DOI:** 10.1186/1752-1947-4-175

**Published:** 2010-06-09

**Authors:** George M Yousef, Manal Y Gabril, Sahar Al-Haddad, Anna Marie Mulligan, R John Honey

**Affiliations:** 1 Department of Laboratory Medicine, and the Keenan Research Centre in the Li Ka Shing Knowledge Institute. St. Michael's Hospital, Toronto, Ontario, Canada; 2 Department of Laboratory Medicine and Pathobiology, University of Toronto, Toronto, Ontario, Canada; 3 Department of Pathology, London Health Sciences Center, University of Western Ontario, London, Ontario, Canada; 4 Division of Urology, Department of Surgery, St. Michael's Hospital Toronto, University of Toronto, Ontario, Canada

## Abstract

**Introduction:**

Invasive lobular carcinoma of the breast represents approximately 6.3% of mammary malignancies. Distant metastasis of invasive lobular carcinoma to the peritoneum or retroperitoneum has been reported fairly frequently.

**Case presentation:**

We report the case of a 59-year-old Caucasian-Canadian woman with invasive lobular carcinoma of the breast presenting with retroperitoneal fibrosis and bilateral ureteral obstruction. Intra-operative pathology consultation did not reveal malignancy. The diagnosis, however, was confirmed on permanent sections by histological appearance in addition to immunohistochemistry. To the best of our knowledge, this is the first reported case of invasive lobular carcinoma of the breast presenting with retroperitoneal fibrosis.

**Conclusion:**

In a case of unexplained ureteric obstruction and retroperitoneal fibrosis, more comprehensive physical examination and additional ancillary studies may be warranted to rule out malignancy as an underlying etiology. This case also emphasizes that intra-operative frozen section consultation cannot always be fully relied upon to exclude a malignancy as the etiology of retroperitoneal fibrosis. Moreover, in permanent histopathology sections, immunohistochemistry testing can be of value to rule out metastatic disease where the morphology is not salient. There is a need for a thorough physical examination of patients with retroperitoneal fibrosis, including the breast and gynecological organs.

## Introduction

Invasive lobular carcinoma (ILC) of the breast represents 6.3% of all breast carcinomas [1]. It differs from invasive ductal carcinoma, the most common type of breast cancer, in its histologic and radiologic appearances, as well as in its pattern of metastases. While spread to lymph nodes, lung and liver are common in both ductal and lobular carcinomas, ILC has been found to frequently metastasize to the gastrointestinal tract, peritoneum and retroperitoneum, and gynecological organs [2-5]. Patients with genitourinary involvement not infrequently develop hydronephrosis [5,6]. However, this typically occurs in the setting of a known diagnosis of primary breast carcinoma.

Retroperitoneal fibrosis (RPF) is an uncommon disease in which dense fibrous tissue proliferates in the retroperitoneum [7]. It is commonly manifested by dull back or flank pain [7,8]. The late manifestations of RPF include encasement of the abdominal aorta and its branches. This fibrotic tissue also frequently encases the ureters causing their compression or obstruction, with secondary hydronephrosis, which may lead to uremia and renal failure [9].

Two thirds of the cases of RPF are considered to be idiopathic [8]. Recent studies point to an autoimmune mechanism in some of these cases [10]. Other causes include infections, previous surgery, drugs, and retroperitoneal hemorrhage. Also, in approximately 8% of cases, RPF is associated with malignancy [9]. This association is observed with lymphomas, sarcomas, and many carcinomas, including metastatic diseases from breast, stomach, colon and lung to the retroperitonum which initiate a desmoplastic reaction resulting in RPF indistinguishable from idiopathic RPF on imaging [11,12].

We report a case of metastatic lobular carcinoma of breast presented as RPF complicated by ureteric obstruction. To our knowledge, this is the first report of lobular carcinoma of breast presenting as RPF.

## Case presentation

A 59-year-old Caucasian-Canadian woman presented with abdominal pain and oliguria. Physical examination revealed an elevated blood pressure and a serum creatinine of 128 umol/L (normal range 42-102 umol/L). An ultrasound examination revealed bilateral hydronephrosis. A non-contrast abdominal computed tomography (CT) scan revealed extensive RPF encasing the ureters without any significant lymphadenopathy. Cystoscopy and ureteric washings were negative for malignancy, bilateral retrograde pyelograms revealed lengthy areas of smooth obstruction with medial deviation of the mid ureters. Bilateral self retaining stents were inserted. A robotic assisted laparoscopic ureterolysis with biopsy of the encasing fibrosis was performed. Tissue was submitted for frozen section consultation that was consistent with RPF. However, the permanent sections were diagnostic for lobular breast carcinoma. Subsequent complete physical examination and full investigations revealed that the presence of a carcinoma of the right breast. Our patient had a remote undocumented history of a benign breast lesion, confirmed by imaging and biopsy, but no details are available about the nature of this lesion.

### Histologic findings

**Figure 1:**
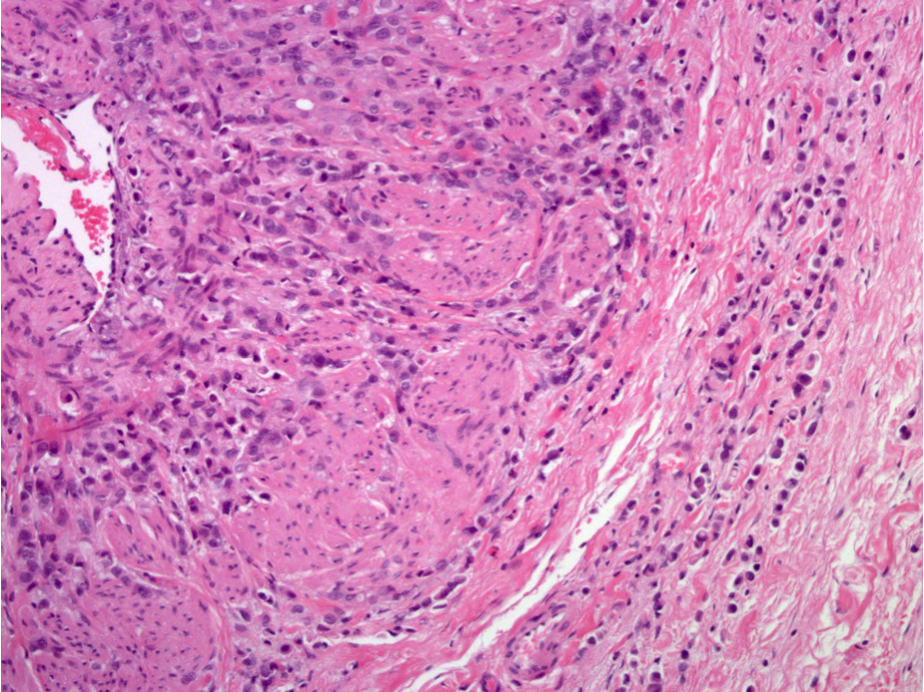
**The histomorphology of the tumor**. (A) Tumor cells infiltrating the wall of the gonadal vein (H & E, original magnification ×200).

**Figure 2:**
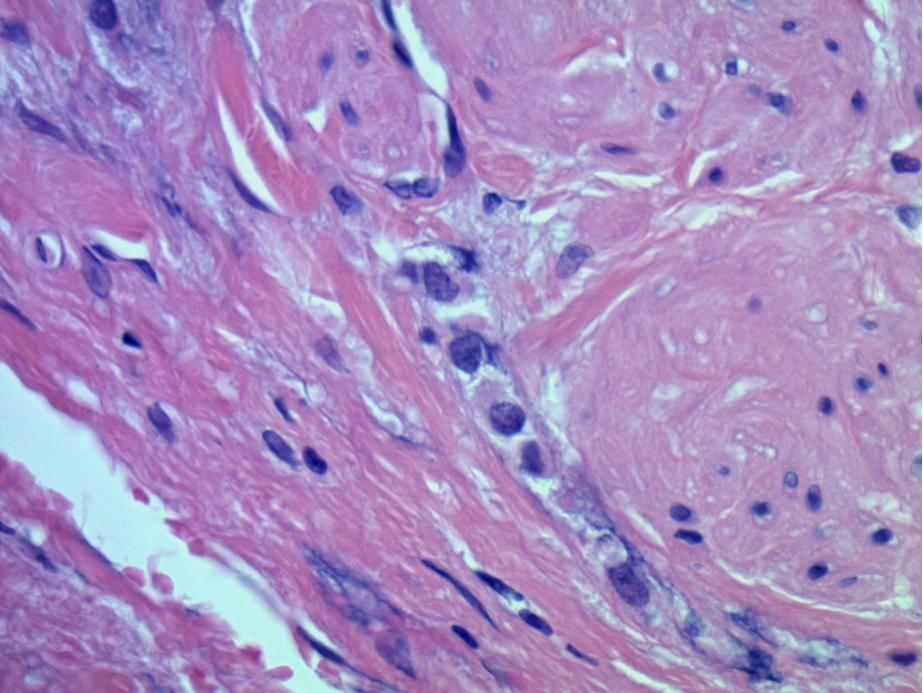
**The histomorphology of the tumor**. Dischohesive cells with single file pattern and intracytoplasmic lumena (H & E, original magnification ×100).

**Figure 3:**
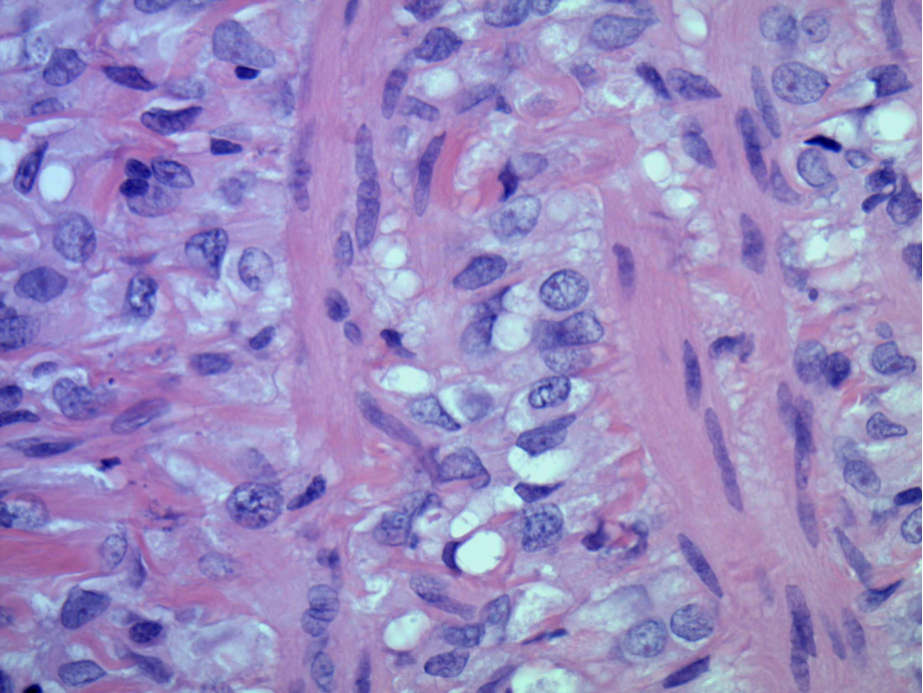
**Higher power showing a uniform population of discohesive cells with small, regular nuclei and inconspicuous nucleoli (H & E, original magnification ×200)**.

Microscopic examinations of the excised retroperitoneal "fibrosis tissue" showed fibrous tissue infiltrated by a discohesive population of small, uniform cells. The gonadal vein showed prominent infiltration of its wall (Figure 1). A single file pattern was identified (Figure 2). Tumor cells showed mild cytological atypia with eccentric nuclei and inconspicuous nucleoli as well as scattered intra-cytoplasmic vacuoles (Figure 3).

**Figure 4:**
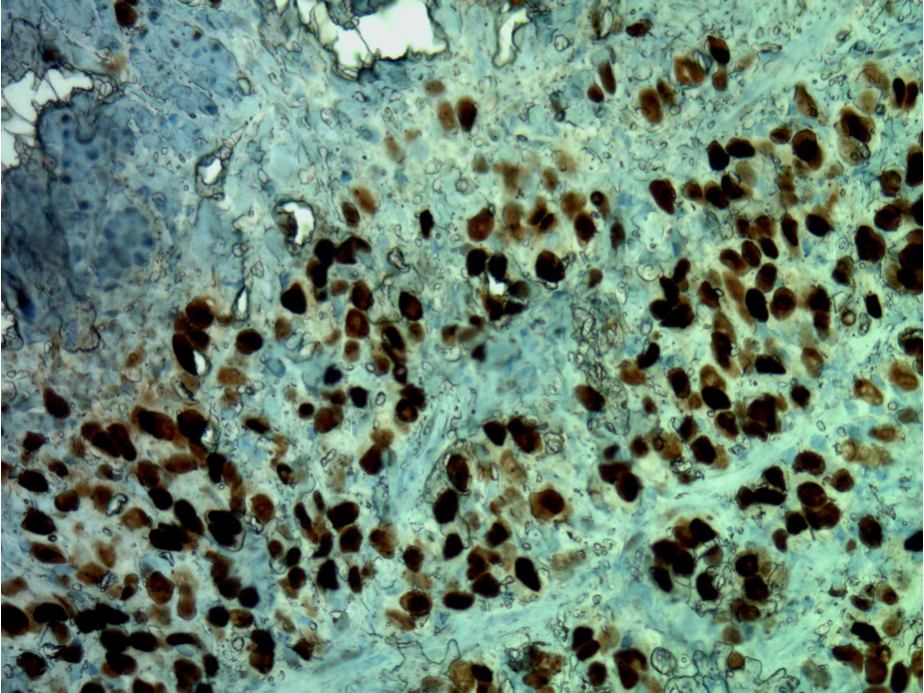
**Immunohistochemistry showing the tumor cells to be positive for estrogen receptors (original magnification ×200)**.

**Figure 5:**
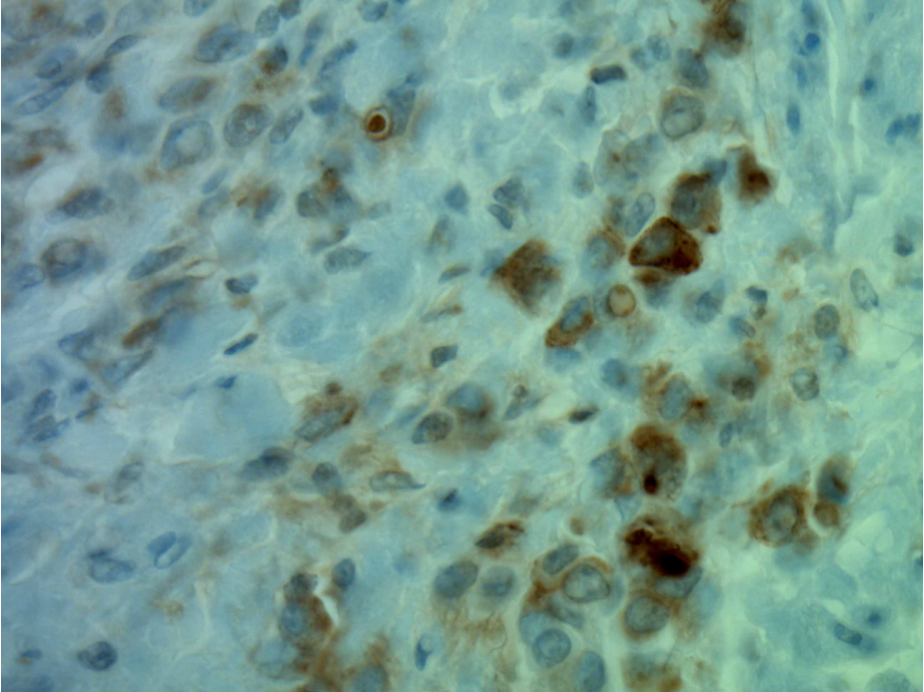
**Immunohistochemistry showing the tumor cells to be positive for GCDFP-15 (original magnification ×200)**.

On immunohistochemistry, tumor cells were positive for low molecular weight and pan-cytokeratin, estrogen receptor (Figure 4), CK7, and GCDFP-15 (Figure 5). Membranous expression of e-cadherin was lost. CK20 was negative. Based on these findings, a diagnosis of metastatic invasive lobular carcinoma of breast origin was made.

## Discussion

Cases of ILC metastasizing to the peritoneal cavity and retroperitoneum are not unusual. Previous reports have shown metastasis to the retroperitoneum complicated by ureteral obstruction [4] but only after the initial diagnosis. To our knowledge, our case is the first report of an ILC of breast with an initial presentation of RPF.

ILC differs from invasive ductal carcinoma of the breast in its morphologic appearance. The former is characterized by a discohesive population of epithelial cells, often of low nuclear grade, without the formation of tubules, nests, sheets or clusters. In this way, this epithelial malignancy may mimic a lymphoid infiltrate; furthermore, ILC is not as frequently associated with desmoplastic response in the adjacent stroma as ductal carcinoma. Effectively, these features make ILC diagnosis in small biopsies or in the setting of a frozen section, as seen in this case, particularly challenging. In fact, in the absence of a clinical history of breast carcinoma, making a diagnosis of metastatic lobular carcinoma at the time of frozen section would be impractical. Immunohistochemical staining with cytokeratins on formalin fixed paraffin embedded tissue can be utilized in subtle cases.

ILC is also distinct in its pattern of metastases, with frequent sites including the gynecologic and gastrointestinal tracts, leptomeninges, orbit, peritoneum and the retroperitoneum. Intra-abdominal involvement typically involves the serosal surfaces and retroperitoneum. Involvement of the latter can result in ureteral obstruction and hydronephrosis is a not infrequent complication. An earlier report documented a series of 24 patients with hydronephrosis from periureteral breast metastases [6]. In a study by Winston *et al*. [5], six patients (11%) had hydronephrosis caused by metastatic ILC to the retropertioneum. It has been suggested that the loss of the cell adhesion molecule, e-cadherin, in ILC may account for these distinct metastatic patterns [13].

RPF is uncommon disease in which dense fibrous tissue proliferates in the retroperitoneum. 60 to 70% of the cases of RPF are idiopathic but other causes include drugs, infection and metastatic carcinomas. The differential diagnosis of cases with clinical and histological presentation simulating RPF should include retroperitoneal lymphoma, sclerosing mesentritis, inflammatory myofibroblastic tumor and some soft tissue sarcomas as liposarcoma [14]. In patients presenting with RPF, a full work up, including a careful physical examination, an abdominal and pelvic CT scan and chest X-ray is mandatory. It is always necessary to exclude metastatic carcinoma as an initiating cause, before diagnosing idiopathic RPF. If a significant retroperitoneal mass is present this may be biopsied under image guidance. In other cases, with the presumed diagnosis of idiopathic fibrosis, a laparoscopic ureterolysis may be performed, releasing the ureters from the fibrosis tissue and wrapping them in omentum. At the time of dissection it is imperative that biopsies are taken from the retroperitoneal tissue to confirm the diagnosis. In our case the frozen section biopsies appeared to confirm the diagnosis of idiopathic RPF and it was not until the final pathology was available that the diagnosis of malignancy was made.

Fibromatosis of the deep tissues often behaves more aggressively than those of superficial sites. In our case, the desmoplastic reaction and consequent fibrous tissue formation, elicited by the tumor is similar to deep fibromatosis. This may make differentiating these two processes very difficult (if not impossible) on clinical/radiologic grounds and a high degree of suspicion of a metastatic neoplasm should be maintained.

The diagnosis was established by the characteristic histologic morphology, in addition to the immunohistochemical profile. ILC are typically positive for cytokeratin 7, ER, and can be positive for GCDFP-15. The majority of ILCs show loss of membranous e-cadherin expression, as seen in this case. The histologic differential diagnosis in this case should include gynecological or gastrointestinal malignancies.

Also, this case emphasizes the fact that a frozen section consultation cannot always be fully relied upon to exclude a malignant etiology of RPF, and negative results can not be conclusive. Immunohistochemistry testing might be of value in this situation, to rule out metastatic disease where the morphology is not salient. Moreover, in the absence of a gross lesion, specimen examination might be better deferred to permanent sections to allow for a more accurate diagnosis.

Another interesting issue to be considered is the inter-observer and sometimes intra-observer variability in diagnosing metastatic lobular breast carcinoma at the time of frozen section. In a series of invasive lobular versus ductal carcinoma metastatic to sentinel lymph nodes, the difference in diagnostic accuracy was not significant [15]. However, in sites where it is not specifically sought histologically, due to processing artefacts and the often low-grade cytology, it could easily be overlooked as chronic inflammation [15].

## Conclusions

In conclusion, we present a case of lobular carcinoma of the breast that presented with RPF and ureteral obstruction. Our case also draws the attention to the need for a thorough physical examination of the patients with RPF including breast and gynecological organs. It also emphasizes that intraoperative consultation (frozen section) might not be reliable to exclude malignant etiology.

## Non-standard abbreviation

ILC: invasive lobular carcinoma; RPF: retroperitoneal fibrosis.

## Competing interests

The authors declare that they have no competing interests.

## Consent

Written informed consent was obtained from the patient for publication of this case report and any accompanying images. A copy of the written consent is available for review by the Editor-in-Chief of this journal.

## Authors' contributions

GMY and MG reviewed the clinical and pathological data and were major contributors in writing the manuscript. SA performed the histological examination of our patient's specimens; RJH analyzed and interpreted our patient's clinical data and laboratory tests; AMM analyzed histological data and performed the immunohistochemical analysis. All authors shared in drafting the manuscript. All authors read and approved the final manuscript.

## References

[B1] Berg JW Hutter RV Breast cancer Cancer 1995 75 257 269 10.1002/1097-0142(19950101)75:1+<257::AID-CNCR2820751311>3.0.CO;2-Y 8001000

[B2] Borst MJ Ingold JA Metastatic patterns of invasive lobular versus invasive ductal carcinoma of the breast Surgery 1993 114 637 641 8211676

[B3] Harris M Howell A Chrissohou M Swindell RI Hudson M Sellwood RA A comparison of the metastatic pattern of infiltrating lobular carcinoma and infiltrating duct carcinoma of the breast Br J Cancer 1984 50 23 30 6331484 10.1038/bjc.1984.135 PMC1976917

[B4] Lamovec J Bracko M Metastatic pattern of infiltrating lobular carcinoma of the breast: an autopsy study J Surg Oncol 1991 48 28 33 10.1002/jso.2930480106 1653879

[B5] Winston CB Hadar O Teitcher JB Caravelli JF Sklarin NT Panicek DM Liberman L Metastatic lobular carcinoma of the breast: patterns of spread in the chest, abdomen, and pelvis on CT AJR Am J Roentgenol 2000 175 795 800 10954469 10.2214/ajr.175.3.1750795

[B6] Grabstald H Kaufman R Hydronephrosis secondary to ureteral obstruction by metastatic breast cancer J Urol 1969 102 569 576 4310510 10.1016/s0022-5347(17)62202-x

[B7] Kottra JJ Dunnick NR Retroperitoneal fibrosis Radiol Clin North Am 1996 34 1259 1275 8898793

[B8] Jones JH Ross EJ Matz LR Edwards D Davies DR Retroperitoneal fibrosis Am J Med 1970 48 203 208 10.1016/0002-9343(70)90116-6 5416262

[B9] Takashima T Onoda N Ishikawa T Koyama T Inaba M Nishizawa Y Nakatani T Wakasa K Hirakawa K Tumor-forming idiopathic retroperitoneal fibrosis: report of a case Surg Today 2004 34 374 378 10.1007/s00595-003-2695-z 15052458

[B10] Kamisawa T Okamoto A IgG4-related sclerosing disease World J Gastroenterol 2008 14 3948 3955 10.3748/wjg.14.3948 18609677 PMC2725332

[B11] Thomas MH Chisholm GD Retroperitoneal fibrosis associated with malignant disease Br J Cancer 1973 28 453 458 4758372 10.1038/bjc.1973.171 PMC2008917

[B12] Wu J Catalano E Coppola D Retroperitoneal fibrosis (Ormond's disease): clinical pathologic study of eight cases Cancer Control 2002 9 432 437 12410182 10.1177/107327480200900510

[B13] Sastre-Garau X Jouve M Asselain B Vincent-Salomon A Beuzeboc P Dorval T Durand JC Fourquet A Pouillart P Infiltrating lobular carcinoma of the breast. Clinicopathologic analysis of 975 cases with reference to data on conservative therapy and metastatic patterns Cancer 1996 77 113 120 10.1002/(SICI)1097-0142(19960101)77:1<113::AID-CNCR19>3.0.CO;2-8 8630916

[B14] Corradi D Maestri R Palmisano A Bosio S Greco P Manenti L Ferretti S Cobelli R Moroni G Dei Tos AP Buzio C Vaglio A Idiopathic retroperitoneal fibrosis: clinicopathologic features and differential diagnosis Kidney Int 2007 72 742 753 10.1038/sj.ki.5002427 17622270

[B15] Horvath JW Barnett GE Jimenez RE Young DC Povoski SP Comparison of intraoperative frozen section analysis for sentinel lymph node biopsy during breast cancer surgery for invasive lobular carcinoma and invasive ductal carcinoma World J Surg Oncol 2009 7 34 10.1186/1477-7819-7-34 19317888 PMC2667517

